# Secondary congenital nephrotic syndrome complicated by acute mesenteric ischemia: A case report 

**DOI:** 10.5414/CNCS111438

**Published:** 2025-03-03

**Authors:** Gita Benbrahim Ansari, Hanane Aboufaris, Zineb Hammoumi, Mounia Al Zemmouri, Kenza Bouayed

**Affiliations:** 1 Department of Pediatric Rheumatology and Nephrology, and; 2 Department of Pediatric Surgery, Mother and Children Hospital Abderrahim Harouchi, University Hospital Center Ibn Rochd, Casablanca, Morocco

**Keywords:** nephrotic syndrome, thrombosis, mesenteric ischemia, CMV, anticoagulation

## Abstract

Thromboembolic events are among the most serious, yet rare complications of nephrotic syndrome. While peripheral venous thrombosis and pulmonary embolism are the most common, superior mesenteric artery thrombosis is a rare but life-threatening occurrence. We present a case of severe cytomegalovirus (CMV) infection complicated by congenital nephrotic syndrome, leading to mesenteric ischemia.

## Introduction 

Congenital nephrotic syndrome (CNS) is a rare condition characterized by the onset of nephrotic syndrome (NS) within the first 3 months of life. The etiology is usually due to mutations in genes coding for protein constituting the glomerular filtration barrier (NPHS1, NPHS2, WT1, LAMB2). Other rare causes include congenital syphilis, toxoplasmosis, and congenital viral infections such as cytomegalovirus (CMV). It usually leads to massive proteinuria and secondary anasarca [1, 2, 3]. Complications arising from severe proteinuria include pro-thrombotic state, arterial or venous thromboembolism, recurrent infections, fluid and electrolyte disturbances, and impaired growth [1, 2]. Thromboembolism is among the most serious complications of NS, resulting from a state of hypercoagulability leading to thrombus formation and obstruction of blood flow [4]. We present a rare case of superior mesenteric artery thrombosis resulting in small bowel ischemia and necrosis in a 3-month-old male infant with severe CMV infection complicated by congenital NS. 

## Case report 

Our patient is a 3-month-old boy, delivered prematurely at 34 weeks of gestation, with a history of congenital CMV infection leading to secondary congenital NS, confirmed by hypoalbuminemia at 1 g/dL, elevated urinary albumin/creatinine ratio at 17.5 g/g, and positive CMV serology IgM at 8.08 Au/mL (normal range < 1 Au/mL), IgG at 45.90 Au/mL (normal range < 6 Au/mL), and polymerase chain reaction at 4,655 UI/mL confirming CMV origin. Treatment consisted of frequent albumin infusions at 1 g/kg per infusion and angiotensin- converting enzyme (ACE) inhibition (enalapril at 0.2 mg/kg/day), resulting in the improvement of ascites and peripheral edema. He also received vitamin D at 600 UI/day and low-dose acetylsalicylic acid at 3 mg/kg/day. Ganciclovir treatment at 5 mg/kg/day was initiated 2 weeks after presentation, following confirmation of CMV infection. After 2 days of antiviral therapy, the child presented with abdominal pain and distension, bilious vomiting, and obstipation. On physical examination, he was afebrile. He had normal systolic blood pressure at 82 mmHg and diastolic blood pressure at 50 mmHg. He weighed 3.5 kg (weight below the 3^rd^ percentile for age and gender). He showed signs of dehydration with sunken eyes and fontanel, dry mouth, pallor, respiratory distress (polypnea at 60 breaths per minute) and tachycardia of 180 beats per minute. The abdomen was distended and tense. 

**Figure 1:**
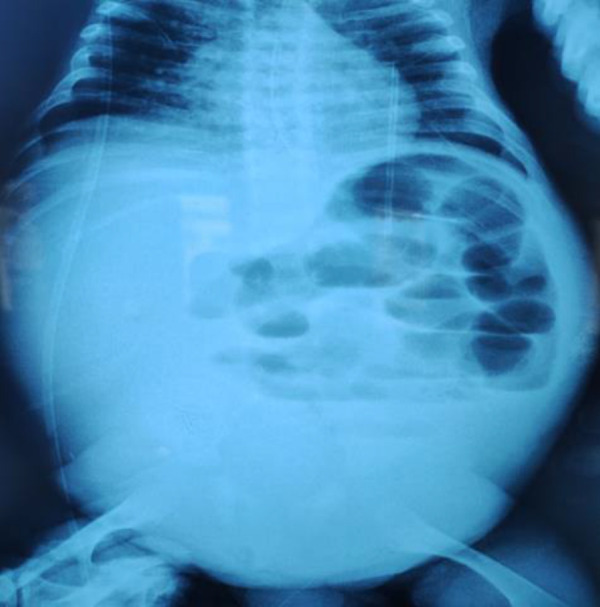
Abdominal X-ray demonstrating a small bowel obstruction (multiple air fluid levels).

Blood investigations showed anemia of 7.6 g/dL, hypoalbuminemia of 11 g/L, urine protein/creatinine ratio of 13.7 g/g, hyponatremia of 129 mmol/L, leukocytosis (leukocytes at 29,000/mm^3^), thrombocytosis of 798,000/mm^3^, and increased C-reactive protein (CRP) of 209 mg/L. The child presented with hypertriglyceridemia of 2.8 g/L, hypercholesterolemia of 3.3 g/L, and elevated LDL level of 2.4 g/L. Abdominal X-ray showed markedly dilated bowel loops and multiple air fluid levels contrasting with the absence of air in the pelvis (Figure 1). 

**Figure 2:**
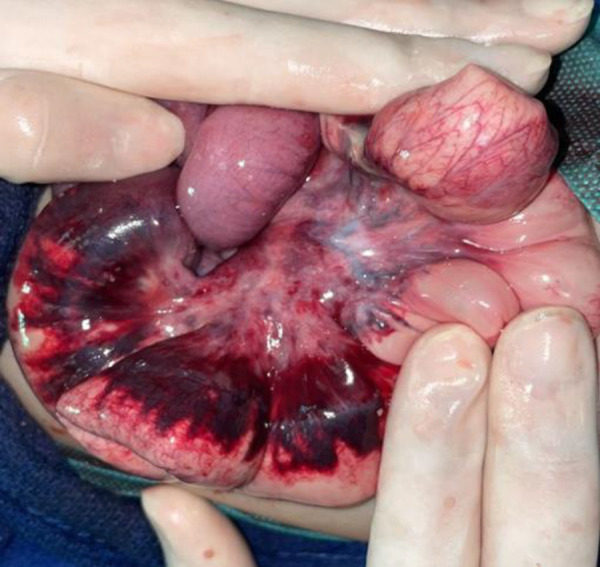
Small bowel necrosis.

Diagnosis of bowel obstruction led to an emergency exploratory laparotomy that revealed ascites and extensive small bowel necrosis, 50 cm upstream of the ileocecal valve, compromising the terminal ileum (Figure 2). The necrotic segment was resected, and an end ileostomy was created. Post-operatively, the patient developed sepsis and was treated with gentamicin, ceftriaxone, and metronidazole. Unfortunately, with his immunodeficient state, the devastating sepsis culminated in the patient’s death. No genetic tests were performed. 

## Discussion 

Thromboembolism is a rare but serious complication of NS [1]. These patients present with severe proteinuria and are at high risk of thrombogenesis [5, 6, 7, 8]. The prothrombotic state in NS is attributed to urinary loss of anticoagulants leading to diminished protein S and C as well as antithrombin III levels. It is also related to increased procoagulant activity and elevated platelet counts. Predisposing factors include abnormalities in platelet activation and aggregation, activation of the coagulation system, increased synthesis of coagulation factors, and accumulation of α2-macroglobulin [5, 7, 9, 10]. 

The incidence of thromboembolic complications in adults with NS ranges from 9 to 70%, whereas their frequency in children is only between 1.8 and 6.6%, according to various studies [1, 4, 6, 7, 9, 10, 11, 14]. However, the frequency is higher in children with CNS, estimated at ~ 10%, and even higher in those with “secondary” CNS, at 17.1% [4]. In fact, several studies have reported a prevalence of thromboembolic complications of 10 – 29% in patients with CNS over their disease course. This variability is partially attributed to the marked genotypic and phenotypic variation in CNS [2]. 

Thrombosis can affect any vessel in patients with NS, but the most commonly affected sites are the deep veins of the leg, followed by the renal vein and pulmonary artery, whereas arterial thrombosis is less common, and literature about it remains scarce [1, 11]. Among arterial thrombosis, superior mesenteric artery thrombosis is a very rare and life-threatening condition [9]. To date, only few cases of acute mesenteric ischemia in patients with NS have been reported, including 3 CNS cases (Table 1). Our case emphasizes this rare and severe condition in NS, which systematically led to death in all congenital NS. The onset of NS before the age of 3 months seems to be a poor prognostic factor. 

Superior mesenteric vein or artery thrombosis usually present with nonspecific symptoms, such as acute and severe abdominal pain, which may be associated with nausea and vomiting [7]. In the early stage, there is a discrepancy between the intensity of the abdominal pain and the absence of signs on physical examination, which delays diagnosis [13]. This finding suggests that imaging examinations must be considered, irrespective of the symptoms, for patients with NS [7]. 

Untreated mesenteric arterial thrombosis often causes acute mesenteric ischemia with intestinal necrosis, as reported in our patient. At an advanced stage, mesenteric thrombosis may be revealed by fever, bloody diarrhea, and shock syndrome with a fatal outcome [12, 16]. Infection, as a common NS complication, can also contribute to death [12]. Our patient died of severe sepsis inherent both to the immunodepression associated with NS and the consequences of intestinal ischemia. 

Thromboprophylaxis in NS is controversial, but prophylactic anticoagulation is recommended for patients at high risk of thromboembolic events, such as those with albumin concentration < 2 g/dL, fibrinogen > 6 g/L, or antithrombin III level < 70% of the normal value [10]. There is limited literature on primary thromboprophylaxis for CNS. To reduce thrombosis risk, management includes reducing urinary protein loss and administering anticoagulant therapies. This can be achieved through bilateral nephrectomy and early dialysis or unilateral nephrectomy with ACE inhibitors. Warfarin, monitored via the international normalized ratio, and enoxaparin, monitored with anti-factor Xa assays, are effective anticoagulants, though careful monitoring is essential due to the bleeding risk at supratherapeutic levels [2]. Aspirin is rarely used as primary thromboprophylaxis in CNS, and unfractionated heparin is unsuitable due to its need for continuous infusion and high side-effect profile [3]. Once a thromboembolic event occurs, treatment involves higher doses of the same anticoagulants for 6 – 12 months or until NS remission [12]. Direct oral anticoagulants have not been investigated for CNS [2]. 

## Conclusion 

Mesenteric ischemia related to thrombosis in the superior mesenteric artery is a rare life-threatening condition. It should be suspected in any NS patient with any acute, nonspecific digestive symptoms to avoid a late diagnosis and allow early management for better prognosis. 

## Informed consent 

Written informed consent was obtained from the patient’s legally authorized representatives prior to his inclusion in this report. 

## Authors’ contributions 

Ghita Benbrahim Ansari: Formal analysis; funding acquisition; investigation; methodology; project administration; resources; software; supervision; validation; visualization; writing – original draft. 

Hanane Aboufaris: Funding acquisition; investigation; methodology; resources; writing – original draft. 

Zineb Hammoumi: Funding acquisition; investigation; methodology; resources; writing – original draft. 

Mounia Al Zemmouri: Funding acquisition; investigation; methodology; resources; writing – original draft. 

Kenza Bouayed: Formal analysis; funding acquisition; investigation; methodology; project administration; resources; software; supervision; validation; visualization; writing – original draft. 

## Funding 

This research received no external funding. 

## Conflict of interest 

The authors declare no conflict of interest. 

**Table 1.** Summary of reported cases of mesenteric thrombosis complicating nephrotic syndrome [9, 12, 13, 14, 15].

	Age	Sex	NS form	Site of thrombosis	Treatment	Outcome
**Patient 1 [** 13 **]**	**19 days**	**Female**	**Congenital NS of the Finnish type**	**Mesenteric arterial thrombosis**	**Surgical treatment + antibiotics**	**Fatal**
Patient 2 [15]	8 years	Male	Steroid-dependent NS	Mesenteric arterial thrombosis	Surgical treatment + antibiotics	Favorable
Patient 3 [9]	7 years	Male	Steroid-dependent idiopathic NS associated with homozygous mutation of methylenetetrahydrofolate reductase	Mesenteric arterial thrombosis	Surgical treatment + steroid pulses	Favorable
Patient 4 [12]	20 years	Female	Steroid-dependent idiopathic NS revealed by cerebral venous thrombosis with pulmonary embolism, at age of eight	Mesenteric arterial thrombosis	Surgical treatment	Fatal
**Patient 5 [** 14 **]**	**18 months**	**Female**	**Congenital NS**	**Superior mesenteric vessels thrombosis**	**Surgical treatment + antibiotics**	**Fatal**
**Our patient**	**3 months**	**Male**	**CMV infection leading to secondary congenital NS**	**Superior mesenteric vessels thrombosis**	**Surgical treatment + antibiotics**	**Fatal**

Bold = cases of congenital nephrotic syndrome. NS = nephrotic syndrome.
